# Selectable phase formation in VAlN thin films by controlling Al^+^ subplantation depth

**DOI:** 10.1038/s41598-017-17846-5

**Published:** 2017-12-13

**Authors:** G. Greczynski, S. Mráz, L. Hultman, J. M. Schneider

**Affiliations:** 1 Thin Film Physics Division, Department of Physics (IFM), Linköping University, SE-581 83 Linköping, Sweden; 2 Materials Chemistry, RWTH Aachen University, Kopernikusstr. 10, D-52074 Aachen, Germany

## Abstract

We report on a thin film synthesis technique which allows for unprecedented control over the crystalline phase formation in metastable transition metal nitride based layers. For the model material system of V_0.26_Al_0.74_N, a complete transition from hexagonal to supersaturated cubic structure is achieved by tuning the incident energy, hence subplantation depth, of Al^+^ metal ions during reactive hybrid high power impulse magnetron sputtering of Al target and direct current magnetron sputtering of V target in Ar/N_2_ gas mixture. These findings enable the phase selective synthesis of novel metastable materials that combine excellent mechanical properties, thermal stability, and oxidation resistance.

## Introduction

Controlled phase formation of transition metal (TM) nitride based coatings deposited by magnetron sputtering, with numerous applications from wear-protective coatings on cutting tools and components in automotive engines, is critical as it directly affects the performance. For example, precipitation of thermodynamically-favored wurtzite-AlN (*w*-AlN) phase in TiAlN-based coatings is well-known to deteriorate mechanical properties^1,2^, while NaCl-cubic structure spinodal decomposition leads to beneficial hardening^1^. The former presents a great challenge in the design of next generations of TM(N)-based coatings with improved thermal and chemical stability, where alloying with Al to obtain ternary single phase NaCl-structure films is a commonly adopted strategy. Growth of TMAl(N) by conventional DC magnetron sputtering, with low-energy inert-gas ion irradiation of the film surface, typically results in moderate solubility levels, which for the well-studied Ti_1*-x*_Al_*x*_N material system are typically *x*_*max*_ ~ 0.50 at growth temperatures *T*_*s*_ = 500 °C^3,4^.

Recently, we used VAlN as a model system to illustrate a new concept for the synthesis of metastable single-phase NaCl-structure thin films with Al content far beyond solubility limits obtained with conventional plasma processes. By employing high-intensity temporal fluxes of Al^+^ metal ions available from high-power pulsed magnetron sputtering (HIPIMS)^5^ source superimposed onto a continuous V neutral flux supplied from a DC-operated target (hybrid Al-HIPIMS/V-DCMS co-sputtering)^6–8^ we demonstrated single-phase NaCl-structure V_1-*x*_Al_*x*_N films (*c*-VAlN) with an unprecedented metastable Al solubility limit of *x*_*max*_ = 0.75^9,10^, significantly higher than *x*_*max*_ = 0.52 obtained with DCMS^11,12^. This was achieved by separating film-forming species in time and energy domains by applying high-amplitude (300 V) low duty cycle (5%) negative substrate bias synchronized with the Al-rich portion of the HIPIMS pulse. The V atoms, predominantly deposited between HIPIMS pulses, reside at the very surface where high adatom mobility and gas-ion-induced mixing drives the system towards thermodynamic equilibrium to form high-VN-content *c*-VAlN crystallites. In contrast, ~300 eV Al^+^ ions, arriving at the film exclusively during the ~100-μs-long metal-ion rich HIPIMS phase, are directly implanted into *c*-VAlN grains buried below the surface, the process we called *subplantation*. As the activation energy for bulk diffusion is larger than for surface diffusion, the mobility on the cation lattice is limited enabling the formation of metastable, supersaturated *NaCl*-VAlN solid solutions. This scenario is in distinct contrast to conventional DC magnetron sputtering, with no time- or energy-separation of sputtered species, in which both V and Al are co-deposited as neutrals, hence coexist at the surface where adatom diffusion is high, resulting in precipitation of the thermodynamically stable *w*-AlN phase as the Al concentration exceeds that of V.

Here, we present direct experimental evidence that the Al subplantation technique allows for a full control over phase formation in VAlN layers. For the V_1-*x*_Al_*x*_N with *x* = 0.74, a complete transition from single-phase wurtzite to supersaturated single-phase NaCl structure is achieved by increasing the implantation depth of Al^+^ metal-ions, which scales with the incident Al^+^ ion energy. The latter is controlled by varying the amplitude of the substrate bias pulse synchronized with the metal-ion-rich portion of HIPIMS flux, using the input from the *in situ* time-resolved ion mass spectrometry measurements performed at the substrate position.

## Methods

**Figure 1:**
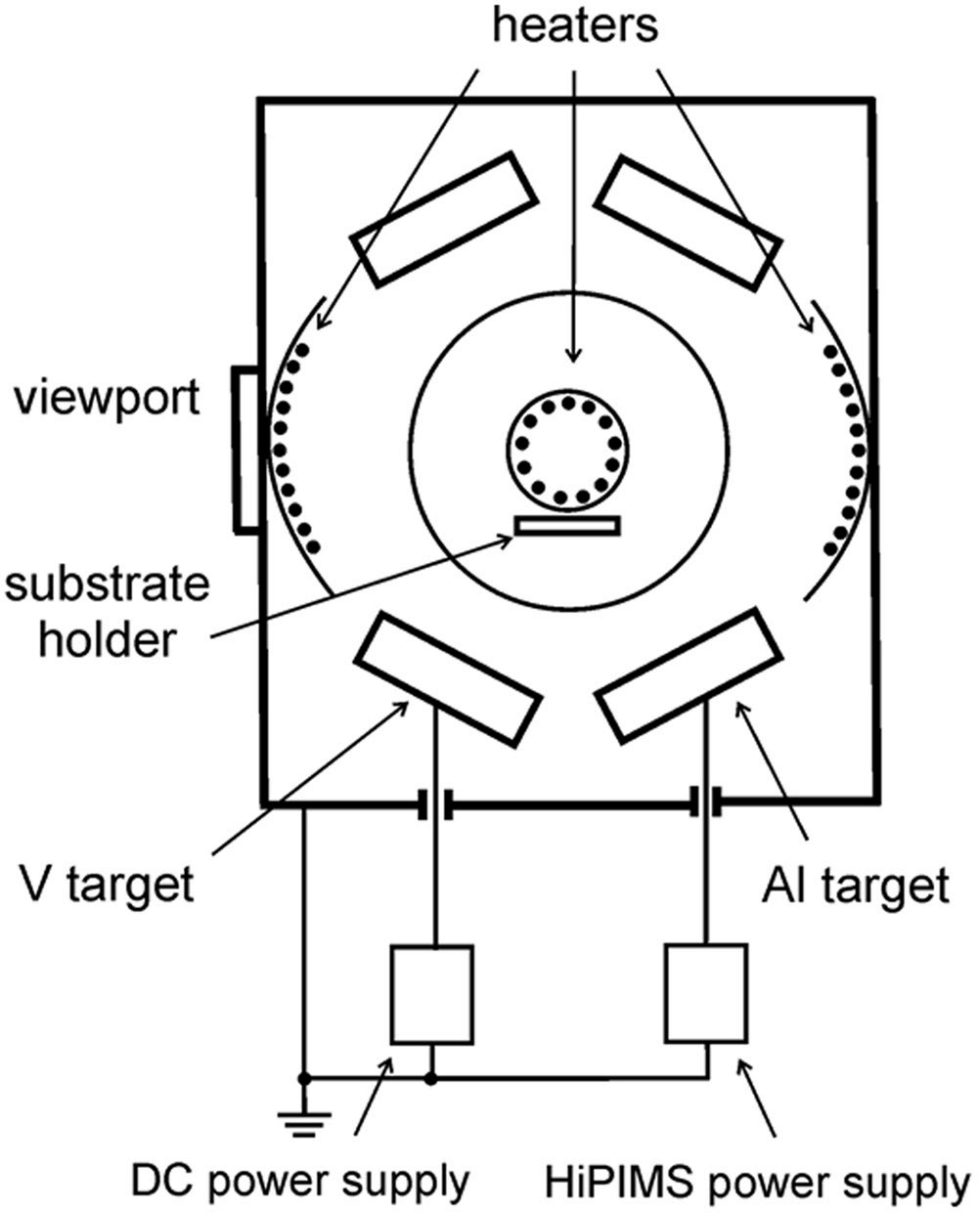
The schematic overview of the deposition setup. Only two cathodes indicated at the bottom are used in the present experiments. All depositions are performed in a stationary configuration.

V_1-*x*_Al_*x*_N films are grown on 1.5 × 2 cm^2^ Si(001) substrates in an industrial CemeCon AG CC800/9 magnetron sputtering system^13^, equipped with Advanced Energy Pinnacle Plus and Melec SIPP2000USB-10-500-S pulser combined with 10 kW ADL GX 100/1000 DC power supply, using V and Al targets assembled from two triangular pieces that form rectangular plates with dimensions 8.8 × 50 cm². Substrates are mounted symmetrically with respect to the targets on a 12 × 31 cm^2^ metal plate arranged in a co-sputtering geometry such that the angle between the substrate normal and the target normal is ~28°, and the target-to-substrate distance is 18 cm (see Fig. 1). The system base pressure is lower than 0.75 mPa (5.63 × 10^−6^ Torr), following the 1 h 40 ± 5 min–long heating step, and the total pressure during deposition is 0.42 Pa (3 mTorr) with a nitrogen flow fraction in the sputtering gas, N_2_/(N_2_ + Ar), varied from 0.29 to 0.32. Substrate temperature *T*_*s*_ during deposition is ~500 °C. The vacuum chamber is vented at a substrate temperature lower than 180 °C to allow for a better control of surface chemistry upon air exposure^14^.

The Al target operates in HIPIMS mode, while the V target is powered by a DC generator (Al-HIPIMS/V-DCMS). The average HIPIMS power is fixed at 2.5 kW (pulsing frequency *f* = 500 Hz, pulse time τ_*HIP*_ = 50 μs), while the power to the DC magnetron is varied from 1.20 to 0.54 kW resulting in V_1-*x*_Al_*x*_N compositions ranging from *x* = 0.53 to 0.80, and the total film thickness between 1490 and 1050 nm. The film growth rates are in the range 16.6 to 11.7 nm/min, or 5.5 × 10^−4^ to 3.9 × 10^−4^ nm per period (1/f), thus several hundred HIPIMS pulses are necessary to grow one monolayer. A negative pulsed substrate bias *V*_*s*_ ranging from floating (on average −15 V) to −400 V, synchronized to each HIPIMS pulse, is used. The bias pulse time τ_*s*_ and an offset τ_*offset*_ are kept constant at 100 and 30 μs, respectively, hence, the resulting bias duty cycle is 5%. Between HIPIMS pulses, the substrate is at floating potential, *V*_*f*_ = −10 V, due to the DC magnetron (with V target) that operates continuously. The floating potential is measured with digital oscilloscope. In addition, V_0.26_Al_0.74_N films are grown with *V*_*s*_ = −300 V using (a) τ_*offset*_ = 50 μs and τ_*s*_ = 30 μs, and (b) τ_*offset*_ = 30 μs and τ_*s*_ = 130 μs.

Compositions of V_1-*x*_Al_*x*_N films are determined by energy-dispersive x-ray spectroscopy (EDX) performed with EDAX instrument attached to JEOL scanning electron microscope JSM-6480 using ToF-ERDA analyzed samples as a standard. Philips X’Pert MRD system operated with point-focus Cu Kα radiation (λ = 1.5418 Å) is used for *x*-ray diffraction (XRD) scans and residual stress analyses by sin^2^ *ψ* technique^15^. The latter is conducted by means of *θ*–2*θ* scans obtained as a function of the sample tilt angle *ψ* defined as the angle between surface normal and the diffraction plane containing the incoming and diffracted *x*-ray beams. *ψ* is varied from 0 to 71.57°, with steps chosen to produce equally-spaced data points on the sin^2^ *ψ* axis. The differential thermal contraction stress correction is calculated using the average thermal expansion coefficient of Si(001) *α* = 2.9×10^−6^ K^−1^ ^16^. Since *α* for V_1−*x*_Al_*x*_N films is unknown, we use a linear extrapolation between *α*_*VN*_ = 9.35×10^−6^ K^−1^ and *α*_*AlN*_ = 8.00×10^−6^ K^−1^ ^17^.

## Results and Discussion

**Figure 2:**
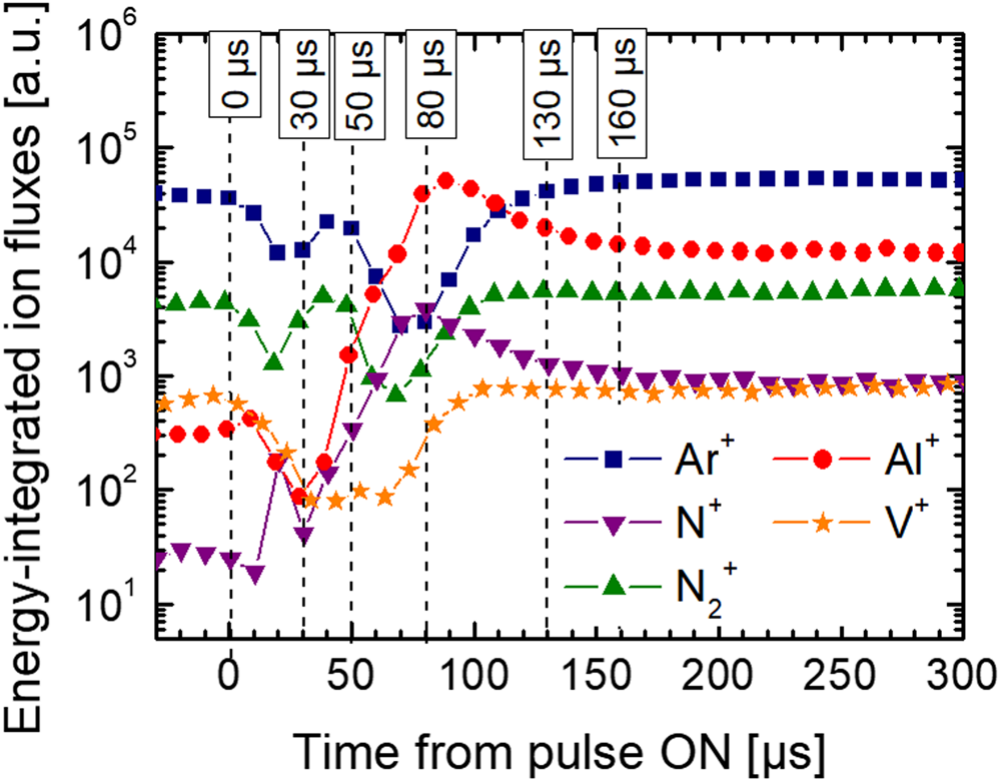
The time evolution of energy-integrated Ar^+^, N_2_^+^, N^+^, Al^+^, and V^+^ ion fluxes detected at the substrate plane during Al-HIPIMS/V-DCMS co-sputtering performed under the same conditions as during film growth. Data are corrected for the ion time of flight inside the mass spectrometer.

The results of *in situ* time-resolved ion mass spectrometry analyses of all major ion fluxes, Ar^+^, N_2_^+^, N^+^, Al^+^, and V^+^, during Al-HIPIMS/V-DCMS performed at the substrate position with plasma process monitor (PPM 422)/mass-energy analyzer (MEA) (Pfeiffer Vacuum) combined with a multi-channel scaler card (MCS-pci, Ortec / Ametek) are shown in Fig. 2. The measurements are carried out at deposition conditions corresponding to the growth of V_0.26_Al_0.74_N film, however, without heating. The Al and V targets are positioned opposite to the MEA orifice in order to adopt the same geometry as during the growth. Ion energy distribution functions (IEDF) are measured as a function of the time-offset with respect to the start of the Al-HIPIMS pulse, from 0 to 500 µs in steps of 10 µs. The time evolution of energy-integrated IEDFs representing the ion fluxes at the substrate plane clearly shows the onset of Al^+^ flux at ~30 µs after the ignition of the Al-HIPIMS pulse, followed by a strong (nearly 3 orders of magnitude) increase to the maximum reached at 90 μs, and a slow decay afterwards. The gaseous Ar^+^ and N_2_^+^ ions experience a severe drop in the intensity during the time period between 10 and 130 µs, which is due to the severe gas rarefaction caused by very high temporal fluxes originating from the HIPIMS source^18–20^. Based on the time evolution of Al^+^ ion flux with respect to that of gas ions, the offset and duration of the bias pulse can be selected in order to precisely synchronize *V*_*s*_(*t*) to the Al^+^-rich portion of each HIPIMS pulse and, hence, insure Al^+^ subplantation.

**Figure 3:**
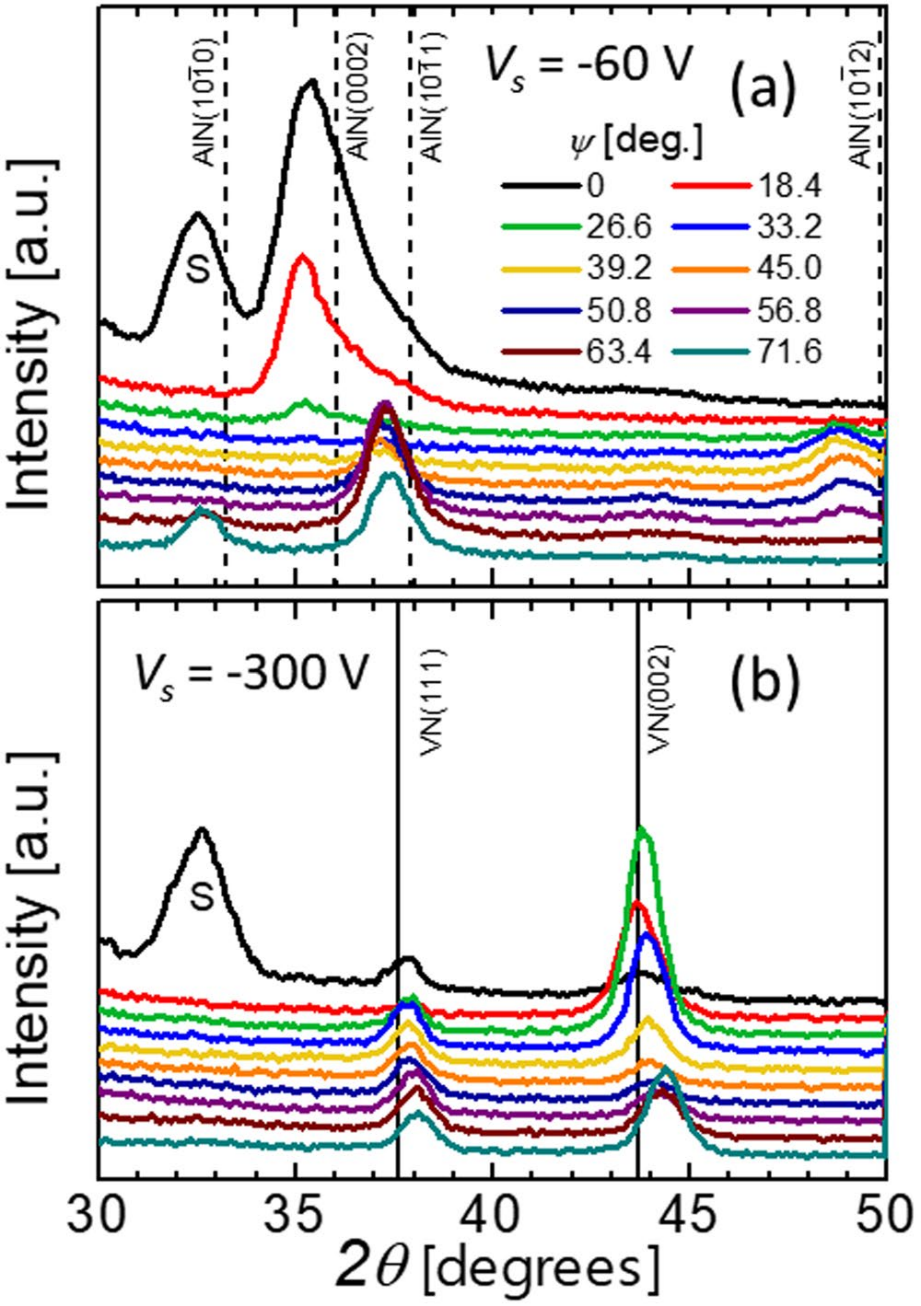
XRD *θ–2θ* scans as a function of the tilt angle *ψ* for V_0.26_Al_0.74_N films grown with the synchronous bias voltage of (**a**) −60 V, and (**b**) −300 V. “S” denotes the forbidden 002 reflection from the Si substrate, which appears due to multiple scattering.

Figure 3 shows the most representative sets of *θ*–2*θ* XRD scans obtained as a function of the sample tilt angle *ψ* defined as the angle between surface normal and the diffraction plane containing the incoming and diffracted *x*-ray beams, for V_0.26_Al_0.74_N films grown with (a) *V*_*s*_ = −60 V, and (b) *V*_*s*_ = −300 V. In both cases, bias pulses are synchronized to the metal-ion rich portion of the HIPIMS discharge, implying the offset of 30 μs and the pulse duration of 100 μs. The only diffraction peaks in the former case are those from *w*-AlN, indicative of a XRD-single-phase film. All wurtzite phase peaks appear at lower 2*θ* angles as compared to the reference *w*-AlN data^21^ indicative of lattice expansion caused by incorporation of larger V atoms. The relaxed lattice constants increase from *a*_0_ = 3.111 Å and *c*_0_ = 4.979 Å for *w*-AlN to 3.160 Å and 5.073 Å, respectively.

In distinct contrast, V_0.26_Al_0.74_N films grown under identical conditions but with *V*_*s*_ = -300 V (Fig. 3(b)) are single phase, with the NaCl structure. The 111 and 002 diffraction peaks recorded at the strain-free angle ψ* are shifted towards higher diffraction angles with respect to the reference VN powder patterns (e.g. 2*θ*_111_ = 37.86° *vs*. 37.61° for powder sample)^22^ due to incorporation of smaller Al atoms into the cubic lattice. The lattice shrinkage is, however, relatively small as compared to the well-studied TiAlN system, due to significantly lower lattice constant of VN (4.139 Å) with respect to that of TiN (4.242 Å)^23^. The relaxed lattice parameter *a*_*o*_ extracted from the 111 and 002 peak positions recorded at ψ = ψ* ^15^ is 4.113 Å. Both 111 and 002 XRD reflections exhibit a shift to higher 2*θ* angles with increasing *ψ*, indicative of compressive residual stresses quantified at −3.8 GPa.

**Figure 4:**
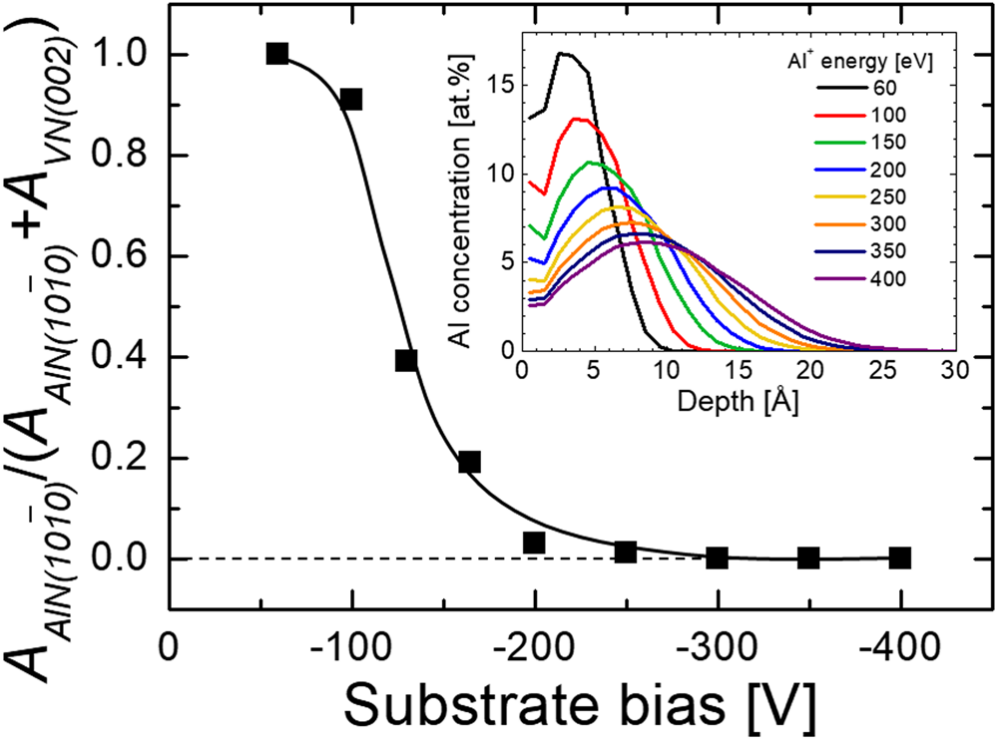
$10\bar{1}0$ *w*-AlN and 002 NaCl *c*-VN peak area ratios obtained from V_0.26_Al_0.74_N films, integrated over all *ψ* angles, and normalized to random powder XRD patterns plotted as a function of the synchronous bias voltage. The insert shows Al^+^ implantation profiles obtained from TRIDYN simulations as a function of Al^+^ energy.

Thus, by varying the amplitude of the synchronous low-duty-cycle bias pulse, while maintaining all other parameters constant, the phase content of V_0.26_Al_0.74_N films can be altered completely from thermodynamically-preferred wurtzite with *V*_*s*_ = −60 V to metastable NaCl-structure obtained with *V*_*s*_ = −300 V. More insight into the underlying physical mechanisms can be gained from the comparison of Fig. 4, where the relative volume fractions χ of *w*-AlN phase, estimated from wurtzite $10\bar{1}0$ and NaCl 002 peak intensities integrated over all *ψ* angles, are plotted as a function of synchronous bias voltage *V*_*s*_. Clearly, with increasing *V*_*s*_, the relative *w*-AlN content decreases rapidly from χ = 1 with *V*_*s*_ = −60 V to 0.91, 0.39, 0.19, and 0.03 for bias amplitude gradually increasing to −100, −130, −165, and −200 V. For |*V*_*s*_| ≳ 250 V, the *w*-AlN fraction is below detection limits. The single phase NaCl structure obtained with higher *V*_*s*_ has been confirmed with selected area electron diffraction in the case of the −300 V sample (not shown). Hence, the critical Al^+^ energy necessary for efficient subplantation in the case of VAlN system is ~200 eV, for the bias pulse synchronized to the metal-rich portion of the HIPIMS discharge. Included as an inset in Fig. 4 are Al^+^ implantation profiles obtained from Monte Carlo TRIDYN simulations of Al^+^ bombardment of VN. Clearly, with increasing Al^+^ incident energy ${E}_{A{l}^{+}}$, the maximum of the implantation profile shifts away from the high-mobility surface layer, defined here as 4 Å which corresponds to about two VN monolayers, and the variation is especially pronounced for ${E}_{A{l}^{+}}$ in the range 60–200 eV. The Al^+^ fraction incorporated below the surface ${\xi }_{A{l}^{+}}$ increases from 27% with ${E}_{A{l}^{+}}$ = 60 eV to 67% with ${E}_{A{l}^{+}}$ = 200 eV. Eventually ${\xi }_{A{l}^{+}}$ = 82% with ${E}_{A{l}^{+}}$ = 400 eV. Note that the first ~1 Å from the surface was not considered in the fraction calculation of the implanted Al^+^.

**Figure 5:**
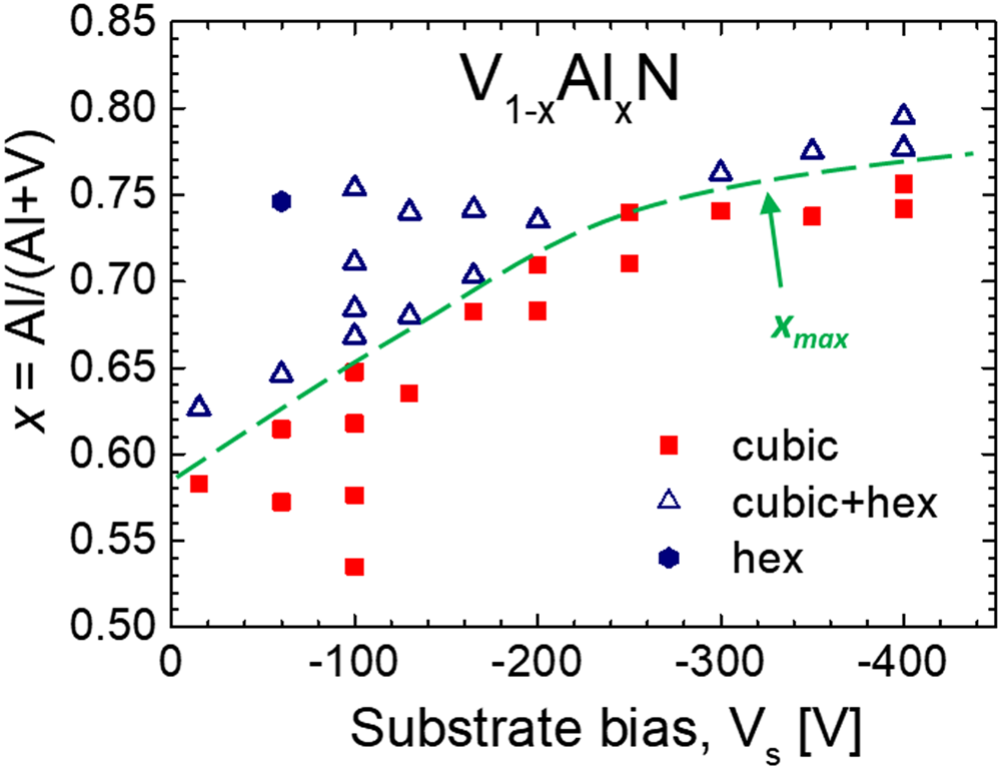
A phase content map for V_1-*x*_Al_*x*_N films with 0.53 ≤ *x* ≤ 0.80, grown with the synchronous negative bias voltage with the amplitude in the range (floating) 15 ≤ |*V*_*s*_| ≤ 400 V. The green dashed curve serves as an eye-guide to the regions of single-phase cubic films (solid red squares) and two-phase layers (open blue triangles) containing both cubic and *w*-AlN grains.

Figure 5 summarizes our findings for the V_1-*x*_Al_*x*_N material system in the form of a phase content map for 0.53 ≤ *x* ≤ 0.80 films grown with the amplitude of the negative bias voltage in the range 15 ≤ |*V*_*s*_| ≤ 400 V. The green dashed curve indicates maximum Al concentration that can be accommodated in the single-phase NaCl structure *x*_*max*_, and serves as an eye-guide to separate the regions of single-phase cubic films (solid red squares) and two-phase layers (open blue triangles) containing both cubic and *w*-AlN grains. *x*_*max*_ clearly increases with increasing the amplitude of the synchronous bias voltage: from 0.59 with *V*_*s*_ = −15 V (floating bias) to 0.66 with *V*_*s*_ = −100 V, 0.72 with *V*_*s*_ = −200 V, 0.75 with *V*_*s*_ = −300 V, and eventually 0.77 with *V*_*s*_ = −400 V. The slope of the *x*_*max*_(*V*_*s*_) curve decreases at higher bias amplitude, revealing saturation of the subplantation effect. This can be understood with the help of TRIDYN simulations (see inset in Fig. 4), which indicate that the largest change to the Al distribution occurs for |*V*_*s*_| < 200 V. In the limit of low *V*_*s*_ amplitude, |*V*_*s*_| < 20 V, *x*_*max*_ approaches the value previously obtained with conventional DCMS processing, *x*_*max*_ = 0.52^10^.

During VAlN film synthesis in the hybrid Al-HIPIMS/V-DCMS configuration, the growth surface is irradiated predominantly by Al^+^ metal-ions during the time period 30–130 μs after the ignition of the HIPIMS pulse, i.e. 5% of the deposition time, as revealed by the ion mass spectrometry analyses performed at the substrate plane (see Fig. 2). Since the cathode sheaths are collision-less and the plasma potential does not exceed a few volts, the incident Al^+^ ion energy is essentially equal to −*eV*_*s*_^24^, as the populations of multiply-charged metal ions is negligible^6^. Between HIPIMS pulses, for 95% of the deposition time, VAlN surface is exposed to a continuous flux of low-energy (~10 eV) gas ions (Ar^+^, N_2_^+^, and N^+^) when the substrate is at floating potential. Since the latter energies are below the lattice displacement threshold (~20–50 eV depending upon the ion and film species involved), the film structure evolution is determined to great extent by the energy and momentum transfer due to metal-ion irradiation during metal-ion-rich HIPIMS phase (both controlled with the amplitude of synchronized bias pulse). Metal-ion bombardment has a clear advantage over gas-ion assisted growth in that metal-ions are incorporated into the structure, while, in contrast, gas-ions are trapped in the interstitial sites^25^ leading to high compressive stress^26,27^ and, as a consequence, to cohesive and/or adhesive film failure^28,29^.

The synchronization of the bias pulse to the Al^+^-rich portion of the HIPIMS discharge enables precise control over the incident ion energy *E*_*i*_ ~ −*eV*_*s*_, and, hence, over the implantation depth of the ionized Al flux (see Fig. 4). This allows for an unprecedented control over the phase formation, as evidenced by XRD data for V_0.26_Al_0.74_N discussed above. At low *V*_*s*_ ~ −15 V, Al^+^ is predominantly deposited at the surface where surface diffusion removes kinetic constraints enabling, in addition to the formation of metastable phases, also nucleation and growth of thermodynamically stable phases. The surface composition is determined by the V atoms deposited predominantly between HIPIMS pulses arriving with a sputter energy of a few eV and the Al flux composed of both ions and neutrals that both end up within the same surface-near region due to similar incident energies. The low-energy (~15 eV) gas ion irradiation due to V-DCMS source enables surface diffusion as discussed above. As long as V flux dominates over that of Al, formation of high V content metastable, cubic VAlN prevails on the surface. Once the Al flux dominates over that of V, the driving force for the formation of thermodynamically stable *w*-AlN phase increases and, eventually, nucleation of the wurtzite phase takes place. With *V*_*s*_ = −20 V this is observed for *x* = 0.58 (see Fig. 5).

With increasing the amplitude of negative substrate bias, the implantation depth for the ionized portion of the Al flux increases and eventually, at −*eV*_*s *_≥ 200 eV, the majority of Al^+^ is subplanted below the high-mobility zone. The composition at the surface is set by the V atoms deposited predominantly between HIPIMS pulses and the *non-ionized* portion of the Al-HIPIMS flux, both arriving with an energy of a few eV. Since a significant portion of the Al flux during Al-HIPIMS is ionized, the Al concentration at the surface is lowered (for constant settings on the Al-HIPIMS source) due to subplantation, preventing the formation of the hexagonal phase (which is otherwise observed) in the high-mobility surface zone. Al^+^ is subplanted directly into the cubic V-rich V_1-*x*_Al_*x*_N matrix triggering local mobility on cation lattice and subsequent quenching, thus enabling the formation of cubic metastable solid solutions. As the activation energy for bulk diffusion is larger than for surface diffusion, the mobility on the cation lattice is limited to few neighboring sites. It appears that the subplantation induced mobility is insufficient to cause nucleation and growth of hexagonal AlN with a larger molar volume than the resulting Al supersaturated *c*-VAlN phase. Eventually, in the limit of |*V*_*s*_| ≳ 250 V, the vast majority of Al^+^ is subplanted below the surface, and *x*_*max*_ is limited by the ionization degree of the Al-HIPIMS flux. The latter is reflected in the *x*_*max*_(*V*_*s*_) plot in Fig. 5 which shows a clear saturation at higher *V*_*s*_ values.

**Figure 6:**
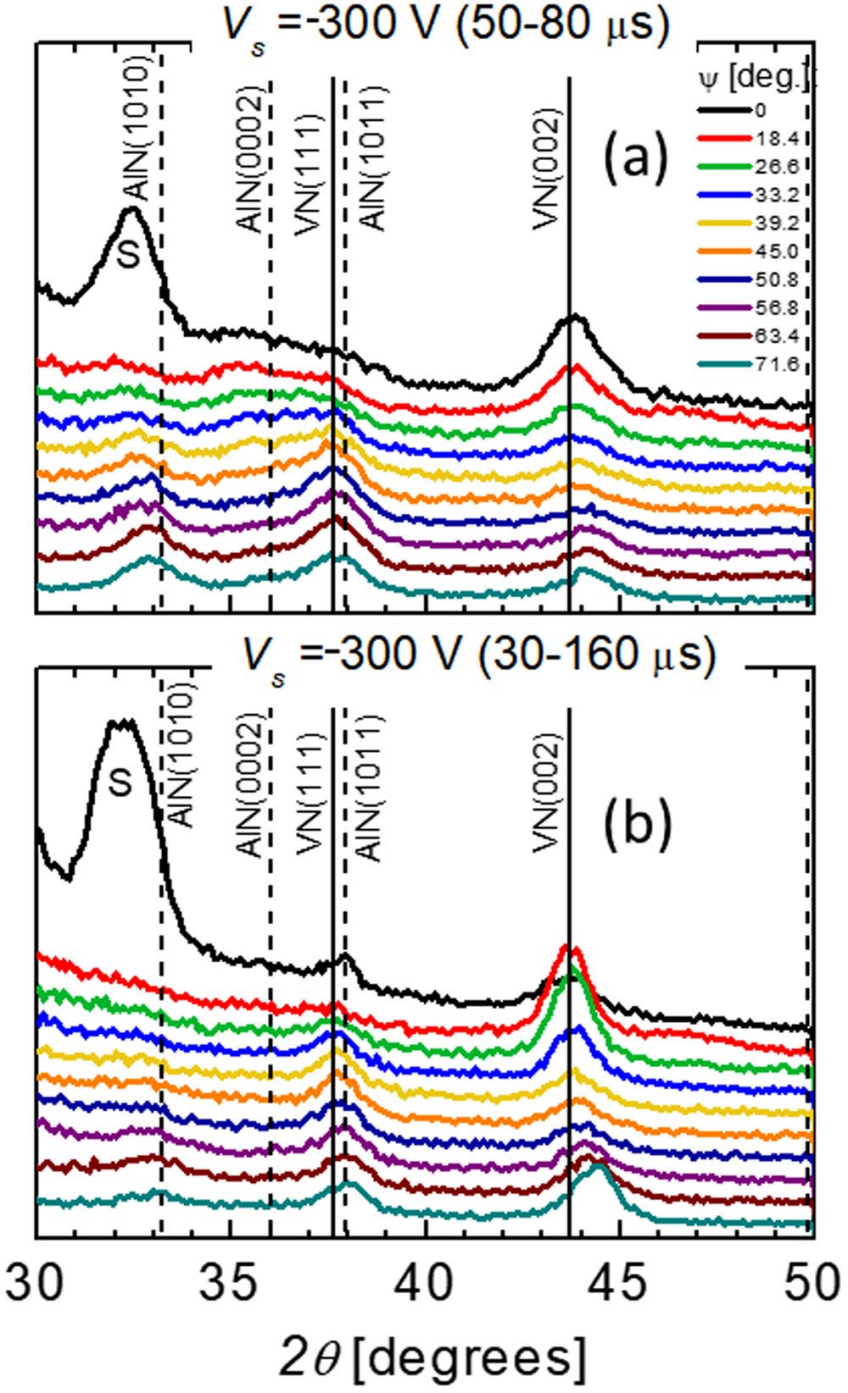
XRD *θ-2θ* scans as a function of the tilt angle *ψ* for V_0.26_Al_0.74_N films grown with the synchronous bias voltage of −300 V and (**a**) 30-μs-long pulses with a 50 μs offset, and (**b**) 130-μs-long pulses with a 30 μs offset. “S” denotes the forbidden 002 reflection from the Si substrate, which appears due to multiple scattering.

Figure 6 presents direct experimental evidence for the critical influence of the bias pulse synchronization (pulse offset and pulse duration) with the Al^+^ portion of the HIPIMS pulse on the phase formation. Two sets of *θ*–2*θ* XRD scans recorded as a function of the sample tilt angle *ψ* are shown for V_0.26_Al_0.74_N films grown with *V*_s_ = -300 V and (a) too short bias pulses of 30 μs applied with an offset of 50 μs, and (b) too long bias pulses of 130 μs starting from 30 μs after ignition of the HIPIMS discharge. In contrast to the single-phase NaCl-structure V_0.26_Al_0.74_N film obtained with 30 μs offset and 100-μs-long bias pulses (see Fig. 3(b)), both layers are two-phase with clear contributions of *w*-AlN. In addition, NaCl-structure diffraction signals exhibit reduced intensity and are broader. In the case of too short bias pulses (Fig. 6(a)), a significant fraction of the Al^+^ flux is not subplanted, and is, hence, deposited onto the very surface where high mobility removes kinetic constraints, resulting in precipitation of *w*-AlN. For too long pulses (Fig. 6(b)), a second phase appears as a consequence of high-energy Ar^+^ (N_2_^+^) bombardment, which^30,31^ induces point defects^32^ that may serve as nucleation sites for the formation of *w*-AlN precipitates at relatively low AlN concentrations.

The here presented experimental evidence clearly illustrates that coating synthesis strategies based on separating ion bombardment of the film forming metallic species in time and energy domains allows for unprecedented enhancements in metastable Al solid solubility in transition metal alumina nitrides with low or moderate compressive stress levels, varying from −1.6 GPa with *x* = 0.53 to −3.8 GPa for *x* = 0.74. Hardness and elastic moduli of Al-HIPIMS/V-DCMS V_1-*x*_Al_*x*_N films with *x* between 0.68 and 0.74 remain high, at respectively ~30 and ~325 GPa. The *H* ^3^/*E* ^2^(*x*) ratio reflecting the materials resistance against plastic deformation^33^ and considered to be an important design criterion^34^ is 0.25–0.30.

Furthermore, a substantial improvement of high temperature oxidation resistance can be expected (based on the significantly enhanced critical Al solubility) which is a significant advantage over conventional processing methods.

## Conclusions

In summary, we present a novel thin film synthesis method, which allows for a complete control over the phase formation in metastable transition metal nitride layers. The technique is based on hybrid high power impulse/dc-magnetron co-sputtering of elemental targets in Ar/N_2_ gas mixture with precise synchronization of the substrate bias pulse to the Al^+^-populated portion of the HIPIMS discharge. For the model material system of V_1-*x*_Al_*x*_N with a high Al content on the cation lattice *x* = 0.74, a complete transition from hexagonal *w*-AlN to supersaturated cubic NaCl structure is achieved by increasing the Al^+^ energy, *ceteris paribus*, by subsequently increasing the subplantation depth of Al^+^ metal-ions, as indicated by Monte Carlo TRIDYN simulations. The former is easily controlled by varying the amplitude of the synchronous low-duty-cycle substrate bias pulse.

### Data Availability

The data and samples analyzed during the current study are available from the corresponding author on reasonable request.

## References

[1] Hörling A, Hultman L, Odén M, Sjölén J, Karlsson L (2005). Mechanical properties and machining performance of Ti1−xAlxN-coated cutting tools. Surf. Coat. Technol..

[2] Kimura A, Hasegawa H, Yamada K, Suzuki T (1999). Effects of Al content on hardness, lattice parameter and microstructure of Ti1−xAlxN films. Surf. Coat. Technol..

[3] Wahlström U (1993). Crystal growth and microstructure of polycrystalline Ti1-xAlxN alloy films deposited by ultra-high-vacuum dual-target magnetron sputtering. Thin Solid Films.

[4] Adibi F, Petrov I, Greene JE, Wahlstrom U, Sundgren J-E (1993). Design and characterization of a compact two-target ultrahigh vacuum magnetron sputter deposition system: application to the growth of epitaxial Ti1-xAlxN alloys and TiN/Ti1-xAlxN superlattices. J. Vac. Sci. Technol. A.

[5] Kouznetsov V, Macak K, Schneider JM, Helmersson U, Petrov I (1999). A novel pulsed magnetron sputter technique utilizing very high target power densities. Surf. Coat. Technol..

[6] Greczynski G (2014). A review of metal-ion-flux-controlled growth of metastable TiAlN by HIPIMS/DCMS co-sputtering. Surf. Coat. Technol..

[7] Greczynski G (2015). Control of Ti1−xSixN nanostructure via tunable metal-ion momentum transfer during HIPIMS/DCMS co-deposition. Surf. Coat. Technol..

[8] Greczynski G (2014). Strain-free, single-phase metastable Ti0.38Al0.62N alloys with high hardness:metal-ion energy vs. momentum effects during film growth by hybrid high-power pulsed/dc magnetron cosputtering. Thin Solid Films.

[9] Greczynski G (2017). Unprecedented Al supersaturation in single-phase rock salt structure VAlN films by Al+ subplantation. J. Appl. Phys..

[10] Greczynski G (2017). Extended metastable Al solubility in cubic VAlN by metal-ion bombardment during pulsed magnetron sputtering: film stress vs subplantation. J. Appl. Phys..

[11] Rovere F (2010). Experimental and computational study on the phase stability of Al-containing cubic transition metal nitrides. J. Phys. D: Appl. Phys..

[12] Zhu P, Ge F, Li S, Xue Q, Huang F (2013). Microstructure, chemical states, and mechanical properties of magnetron co-sputtered V1−xAlxN coatings. Surf. Coat. Technol..

[13] http://cemecon.de/coating_technology/coating_units/cc800_hipims/index_eng.html; accessed in (2017).

[14] Greczynski G, Mráz S, Hultman L, Schneider JM (2016). Venting temperature determines surface chemistry of magnetron sputtered TiN films. Appl. Phys. Lett..

[15] Birkholz, M. *Thin Film Analysis by X-ray Scattering*, Ch. 6, 239–295 (Wiley-VCH, 2006).

[16] Watanabe H, Yamada N, Okaji M (2004). Linear thermal expansion coefficient of silicon from 293 to 1000 K. Int. J. Thermophys..

[17] Pierson, H. O. *Handbook of refractory carbides and nitrides: properties*, *characteristics*, *processing*, *and applications* (William Andrew, 1996).

[18] Horwat D, Anders A (2010). Compression and strong rarefaction in high power impulse magnetron sputtering discharges. J. Appl. Phys..

[19] Anders A (2011). Discharge physics of high power impulse magnetron sputtering. Surf. Coat. Technol..

[20] Britun N, Konstantinidis S, Snyders R (2015). An Overview on Time‐Resolved Optical Analysis of HiPIMS Discharge. Plasma Processes and Polymers.

[21] Kohn JA, Cotter PG, Potter RA (1956). Synthesis of aluminum nitride monocrystals. American Mineralogist.

[22] Natl. Bur. Stand. (U.S.) *Monogr*. **25**, 21, 130 (1984).

[23] Wong-Ng W (1987). Standard X-ray diffraction powder patterns of fifteen ceramic phases. Powder Diffraction.

[24] Anders A (2002). Atomic scale heating in cathodic arc plasma deposition. Appl. Phys. Lett..

[25] Hultman L, Sundgren J‐E, Greene JE (1989). Formation of polyhedral N2 bubbles during reactive sputter deposition of epitaxial TiN (100) films. J. Appl. Phys..

[26] Davis CA (1993). A simple model for the formation of compressive stress in thin films by ion bombardment. Thin Solid Films.

[27] Ulrich S, Theel T, Schwan J, Ehrhardt H (1997). Magnetron-sputtered superhard materials. Surf. Coat. Technol..

[28] Teixeira V (2001). Mechanical integrity in PVD coatings due to the presence of residual stresses. Thin Solid Films.

[29] Oettel H, Wiedemann R (1995). Residual stresses in PVD hard coatings. Surf. Coat. Technol..

[30] Petrov I, Hultman L, Helmersson U, Sundgren J-E, Greene JE (1989). Microstructure modification of TiN by ion bombardment during reactive sputter deposition. Thin Solid Films.

[31] Petrov I, Barna PB, Hultman L, Greene JE (2003). Microstructural evolution during film growth. J. Vac. Sci. Technol. A.

[32] Music D (2017). Correlative plasma-surface model for metastable Cr-Al-N: Frenkel pair formation and influence of the stress state on the elastic properties. J. Appl. Phys..

[33] Tsui TY, Pharr GM, Oliver WC (1995). Nanoindentation and nanoscratching of hard carbon coatings for magnetic disks. Mater. Res. Soc. Symp. Proc..

[34] Musil J (2000). Hard and superhard nanocomposite coatings. Surf. Coat. Technol..

